# E3 Ubiquitin Ligase Regulators of Notch Receptor Endocytosis: From Flies to Humans

**DOI:** 10.3390/biom12020224

**Published:** 2022-01-27

**Authors:** Raluca Revici, Samira Hosseini-Alghaderi, Fabienne Haslam, Rory Whiteford, Martin Baron

**Affiliations:** School of Biological Sciences, Faculty of Biology, Medicine and Health, University of Manchester, Michael Smith Building, Manchester M13 9PT, UK; raluca.revici@postgrad.manchester.ac.uk (R.R.); samira.hosseini@manchester.ac.uk (S.H.-A.); fabienne.haslam@postgrad.manchester.ac.uk (F.H.); rory.whiteford@manchester.ac.uk (R.W.)

**Keywords:** Notch, deltex, NEDD4-family, Suppressor of deltex, endocytosis, signalling, ubiquitin ligase, cell fate, T-cells

## Abstract

Notch is a developmental receptor, conserved in the evolution of the metazoa, which regulates cell fate proliferation and survival in numerous developmental contexts, and also regulates tissue renewal and repair in adult organisms. Notch is activated by proteolytic removal of its extracellular domain and the subsequent release of its intracellular domain, which then acts in the nucleus as part of a transcription factor complex. Numerous regulatory mechanisms exist to tune the amplitude, duration and spatial patterning of this core signalling mechanism. In *Drosophila*, Deltex (Dx) and Suppressor of dx (Su(dx)) are E3 ubiquitin ligases which interact with the Notch intracellular domain to regulate its endocytic trafficking, with impacts on both ligand-dependent and ligand-independent signal activation. Homologues of Dx and Su(dx) have been shown to also interact with one or more of the four mammalian Notch proteins and other target substrates. Studies have shown similarities, specialisations and diversifications of the roles of these Notch regulators. This review collates together current research on vertebrate Dx and Su(dx)-related proteins, provides an overview of their various roles, and discusses their contributions to cell fate regulation and disease.

## 1. Introduction

**Figure 1:**
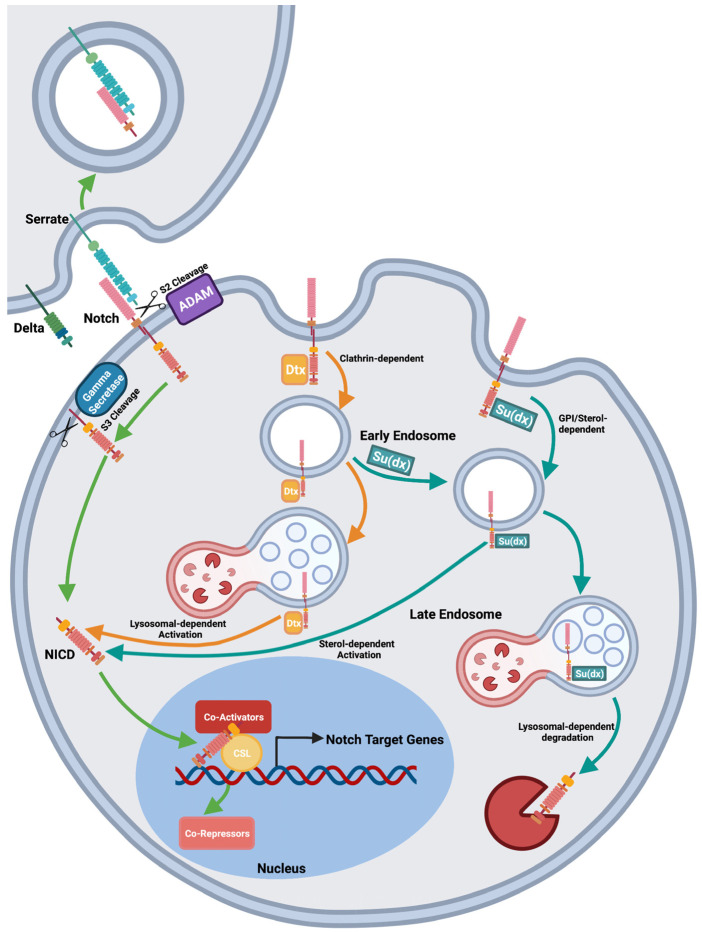
Pathways of Notch activation. Notch is activated by ligand-dependent or independent mechanisms [17]. At the cell surface Notch binds membrane-bound ligands. Endocytic-dependent force generation is thought to cause a conformational change that exposes the S2 cleavage site to ADAM10 dependent proteolysis, which sheds the extracellular domain. The remaining membrane tethered portion is cleaved by gamma-secretase to release the intracellular domain, which relocates to the nucleus and binds to the transcription factor CSL (CBF/Su(H)/Lag2) and coactivators such as Mastermind, to activate Notch target genes. Notch can also be activated by ligand-independent mechanisms following endocytosis of full-length receptor. In *Drosophila* cells, ligand-independent activation can occur following endocytosis by clathrin-dependent or independent routes which are promoted by Dx and Su(dx), respectively, the latter route being marked by the presence of Glycosylphosphatidylinositol (GPI)-anchored proteins. The clathrin-dependent route requires late endosomal/lysosomal fusion and is independent of ADAM10, while the GPI-marked route is independent of lysosomal fusion, and dependent on ADAM10, membrane sterol and glyco-sphingolipids. HECT domain activity opposes both mechanisms of ligand-independent Notch activation by promoting Notch transfer to late endosomal intraluminal vesicles and hence Notch degradation. Both Dx and Su(dx) promoted endocytosis can oppose ligand-induced Notch signalling by removing Notch from the cell surface and denying access to ligand-stimulation.

The Notch pathway is an evolutionarily conserved signalling mechanism with crucial roles in many cellular processes, such as proliferation [1,2,3], differentiation [4,5] and apoptosis [6]. The core pathway is centred around the single-pass transmembrane protein Notch, which is proteolytically processed to release its intracellular domain (ICD) into the cytoplasm. The Notch ICD then relocates to the nucleus and binds to and activates the CSL (CBF/Su(H)/Lag2) transcription factor [7,8]. Signalling can be initiated through two distinct mechanisms. The canonical pathway (Figure 1) requires ligand binding to the Notch receptor in order to expose cleavage sites and trigger subsequent signal activation by ADAM10 (A Disintegrin and metalloproteinase domain-containing protein 10)-dependent cleavage to remove the extracellular domain (ECD) followed by Presenilin-dependent release of the ICD [9]. The ICD relocates to the nucleus where it acts in a complex with the transcription factor CSL. The binding of ICD displaces corepressors from CSL such as Hairless, and recruits coactivators such as Mastermind. Recent work has indicated that nucleo/cytoplasmic shuttling of CSL, regulated by cofactor binding [10,11] also determines its availability in the nucleus (not depicted in Figure 1). In the non-canonical activation pathway (Figure 1), the full-length receptor is endocytosed from the plasma membrane and processing occurs as it travels through the endocytic network [12]. In *Drosophila melanogaster*, two ubiquitin ligase proteins, Deltex (Dx) and Suppressor of deltex (Su(dx)), have been found to regulate endocytic trafficking, ligand-independent signalling and localisation of Notch through clathrin-dependent and independent routes, respectively [13,14,15,16,17]. Both proteins are E3 ubiquitin ligases that interact with the Notch cytoplasmic domain. Dx is a RING (really interesting new gene) finger class E3 ligase and Su(dx) is a HECT (Homologous to E6AP C-terminus) domain ubiquitin ligase of the NEDD4 family. *Drosophila* Nedd4 also acts to down regulate Notch through endocytosis and may have some overlapping function with Su(dx) [16,18]. A critical regulatory node for the ligand-independent Notch activation is whether Notch is retained on the endosome membrane with the ICD exposed to the cytoplasm or whether the full-length Notch is transferred to the intraluminal vesicles. If retained on the endosome membrane, then Notch can be activated following removal of the ECD. This can be an ADAM10-dependent or independent mechanism, depending on if it takes place within the clathrin-independent or clathrin-dependent endocytic pathways, respectively [17].

While research into the roles of Dx and Su(dx) in *Drosophila* has identified different mechanisms by which these proteins can both positively and negatively impact on Notch signalling, less is known about the roles of vertebrate homologues. Recent studies have shown that certain aspects of the activities of vertebrate Dx and Su(dx) homologues on Notch are conserved, while gene duplication of both these regulators and of Notch has enabled specialisation of function. This review will summarise research on *Drosophila* Dx and Su(dx), and then discuss the roles of close vertebrate homologues and their emerging links to disease.

## 2. Lessons from the Fly: Deltex and Su(dx) Act as Both Positive and Negative Regulators of Notch

The *deltex* (*dx*) gene was first implicated in the Notch pathway through genetic interactions, described in *Drosophila*, by which the *dx* mutant was found to rescue a lethal interaction between two *Abruptex*, gain of function, Notch alleles [19]. Expression studies in vitro and in vivo showed that the Dx protein interacts with the Notch ankyrin repeats, a highly conserved domain involved in signalling [14,20]. Later studies showed that Dx binds Notch through its N-terminal WWE domains to form a heterodimer complex stabilised by electrostatic forces [21]. The role of Dx was initially inferred from genetic and phenotypic analysis in *Drosophila*. Several groups have reported that Dx overexpression and activated Notch generate similar phenotypes, while *dx* mutations closely resemble Notch loss of function and include wing margin notches, wing vein thickening, altered ocelli spacing and reduced size of the germline stem cell niche of the ovary [14,20,22,23]. The generation of a *dx* null mutant confirmed that the Dx protein is required only in certain tissues, and that it is not essential for embryo development [24]. However, embryo developmental phenotypes in *dx* maternal/zygotic null embryos have been observed with incomplete penetrance [16,17]. Furthermore, the viability of flies with reduced copy number of key Notch core components is highly sensitive to the additional mutation of *dx* [19,22,24]. These findings suggest that Dx protein plays a role in stabilising, tuning and buffering Notch activity with varying degrees of necessity across different tissues.

Overexpression of Dx was found to promote ligand-independent Notch activation [16,25] associated with apical depletion of Notch in epidermal cells and accumulation of the receptor in endocytic vesicles, while also increasing the half-life of Notch found in these structures. Furthermore, Dx-induced Notch signalling is dependent on the function of components of the trafficking machinery, such as members of the HOPS (homotypic fusion and vacuole protein sorting) and Adaptor-protein 3 complexes [16]. These findings led to a model that Dx regulates Notch by delivering it to the limiting membrane of the late endosome. There, it prevents internalization of the full-length receptor into the intraluminal vesicles of the endolysosome. Since this mechanism of activation is independent of ADAM metalloproteases then it is proposed that removal of the ECD is by the action of lysosomal proteases after lysosomal fusion [16].

As a testament to the intricacy of the Notch signalling regulation, inhibitory Dx roles have also been described. The coexpression of Dx with Kurtz, the fly homologue of the mammalian non-visual β-arrestin, targeted Notch for degradation, and this was correlated with a switch from mono to poly ubiquitination of Notch [26] although a requirement for this Notch ubiquitination by Dx has not been formerly established. Interestingly, coexpression of Dx and Su(dx) also switches the contribution of Dx towards it acting as a negative regulator of Notch [27], and this bipolar behaviour of Dx is revealed by the different Notch signalling outcomes exhibited by *dx* mutants, in different fly tissues, depending on copy number variation of the Su(dx) gene [17]. Mutants of the latter also show a bipolar behaviour to up or down-regulate Notch. When raised at high temperature, Su(dx) mutant flies exhibit a gain of function Notch signal, denoted by gaps in the vein patterning of the fly wing, revealing its role as a negative regulator. However, at low temperature, Su(dx) acts as a positive regulator of Notch and this switch in function is due to temperature-dependency of its HECT domain ubiquitin ligase [17]. The activity of the latter is required to transfer Notch from the endosomal membrane into intraluminal vesicles to switch off Notch activity. Endosomal Notch can therefore act as a buffer for overall Notch signalling levels to compensate for environmental temperature-dependent changes to the level of the ligand-dependent signalling component [17].

## 3. Vertebrate Deltex Proteins

### 3.1. Domain Structure of Deltex Proteins

**Figure 2:**
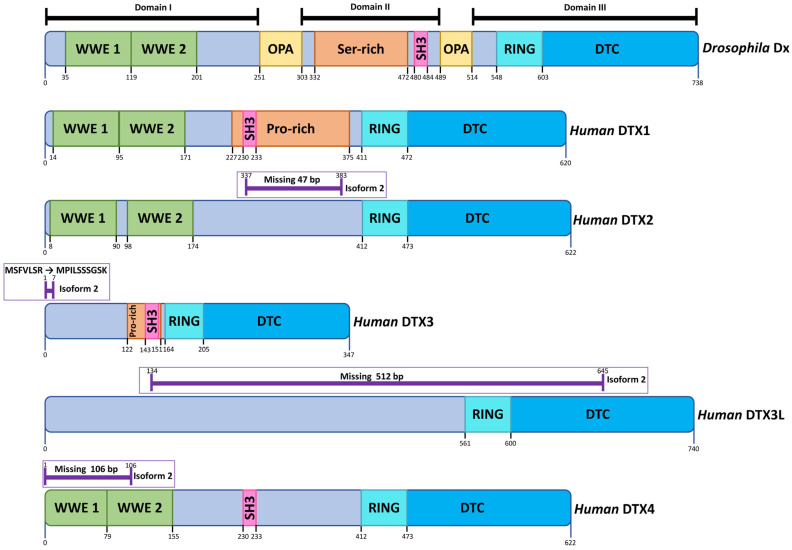
Human Deltex Isoforms. DTX proteins all have in common a conserved C-terminal region comprising a RING domain E3 ubiquitin ligase and a DTC domain. *Drosophila* Deltex can be divided into three subdomains (I–III) separated by poly Q rich regions (OPA). DTX1, DTX2, DTX4 and *Drosophila* Deltex have in common 2 WWE domains, which are Notch-binding regions that interact with the Ankyin domain region of the Notch ICD. Proline-rich regions, in some cases including the PPLP motif that comprises SH3 binding sites, are indicated where present. *Drosophila* Deltex additionally has Q-rich repeat regions (OPA repeats), which separate the N- and C-terminal domain regions from the central Serine-rich region. Variations arising from alternative splicing are indicated on the figure with horizontal purple lines (https://www.uniprot.org, accessed on 18 October 2021) [33].

The structure of *Drosophila* Dx (Figure 2) is split into three domains (I, II and III), separated by glutamine-rich regions named OPA repeats [14,28]. Domain I is a globular region which contains two WWE domains characterised by two conserved tryptophan residues and one conserved glutamate residue [29]. These WWE motifs are responsible for binding to the ankyrin repeats of Notch [21]. Domain II consists of a proline-rich SH3-binding motif (PPLP), whilst the most conserved region of Dx, Domain III contains a RING-H2 zinc-finger domain [30] followed by a Deltex carboxy-terminal (DTC) domain [31]. Though there is only one Deltex protein in *Drosophila*, there are five known Deltex paralogues in humans (referred to as DTX 1, 2, 3, 3L and 4) which are all characterised by the RING-H2 zinc-finger domain in the C-terminus. The identification of the RING-H2 zinc-finger domain enabled the Deltex family to be classified as E3 ubiquitin ligases which catalyse the transfer of ubiquitin from E2 ubiquitin-conjugating enzymes to specific substrates but are also capable of autoubiquitination [32].

The five human Deltex paralogues (Figure 2) differ in the presence of the WWE and SH3-binding motifs. All Deltex paralogues except for DTX3 and DTX3L have two tandem WWE motifs [33]. DTX1, DTX3 and DTX4 contain a PPLP, SH3-binding motif that, in *Drosophila*, has been shown to bind to GRB2 (growth factor receptor binding protein 2), an adaptor protein that positively regulates Ras signalling [30,33,34]. All five human DTX proteins contain the RING-H2 zinc-finger domain followed by the DTC domain [31]. The latter contains a pocket that binds to poly-ADP-ribose-bound proteins targeting them for ubiquitination by the RING-H2 zinc-finger domain [35].

In addition, DTX 2, 3, 3L and 4 are known to each have a second isoform created through alternative splicing (Figure 2). However, little is known about the differences in functional activity between the isoforms. The DTX2 isoform is missing 47 residues, which contain several post-translational modification sites (phosphorylation, acetylation and ubiquitination sites) that may affect how Deltex function is regulated [33,36]. The DTX3 isoform differs in the first seven residues (MSFVLSR→MPILSSSGSK); however, it is unclear how this affects DTX3 function. The DTX3L isoform is missing 512 amino acids (134–645) encompassing the RING-H2 zinc-finger domain thus, removing many post translational modification sites as well as its activity as an E3 ubiquitin ligase [33,36]. The DTX4 isoform is missing the first 106 residues containing the WWE1 motif and the first 12 amino acids of the WWE2 motif which can be predicted to affect the binding of DTX4 to its target substrate [33]. The differences in domain structure may reflect distinctive functions of DTX paralogues both dependent and independent of Notch.

Currently, there are eight known zebrafish DTX paralogues 1, 2, 3, 3L, 3L-2.2, 4a, 4b, and si:dkey-3h3.3). All paralogues apart from 3L contain the highly conserved RING-H2 zinc-finger; however, they differ in the number of WWE motifs. Whilst *Drosophila* Dx, human and mouse DTX1 contain two tandem WWE motifs, zebrafish DTX1 has only one WWE motif [33,37]. DTX 2, 4a and 4b have two tandem WWE motifs whereas DTX3, 3L, 3L-2.2 and si:dkey-3h3.3 do not contain any WWE motifs [33]. None of the zebrafish DTXs contain the PPLP SH3-binding motif although all paralogues contain disordered regions with either largely polar regions, proline-rich regions or polyampholyte regions [33]. Little research has so far been conducted on zebrafish DTX proteins; however, the transparency of zebrafish embryos makes them a useful model organism to study the physiological roles of DTX proteins in development.

### 3.2. DTX1

Extensive evidence supports the conservation of the Dx-Notch interaction between invertebrates and vertebrates. As with *Drosophila* Dx, mammalian DTX proteins interact with the Notch receptor through the conserved WWE domains and ankyrin repeats [34]. It was shown that DTX1 could interact with ICD constructs of two human homologs, NOTCH1 and NOTCH2, as well as enhance transcription of a Notch target gene when co-expressed with Notch ICD constructs in S2 cells [34]. Expression analysis in the human embryo demonstrated that although the DTX1 gene was ubiquitously expressed, mRNA hybridization was especially strong in certain tissues, such as blood vessels, the nervous system, lungs and the mucosal epithelium of the digestive tube [34]. Moreover, a recent study has indicated that DTX1 regulates NOTCH1 by altering its endocytic trafficking [38]. DTX1 inhibits NOTCH1 recycling to the plasma membrane independently of direct ubiquitination of the receptor, which reduces the likelihood of activation events by precluding access of NOTCH1 to cell surface ligands. In this context, DTX1 acts to down regulate NOTCH1 activity. Other studies have indicated that DTX1 can act positively to promote Notch signalling. DTX1 was found to be required for a non-canonical NOTCH1 activation mechanism dependent on the interaction of NOTCH1 with an alternative ligand called F1/Contactin, promoting oligodendrocyte differentiation and proliferation of neural precursor cells [39,40]. The nature of this pathway of activation, and if it is related to the endocytic dependent activation of Notch by Dx in *Drosophila*, is not known. Human NOTCH1 has also been proposed to be activated, independently of ligands, downstream of T-Cell activation, by an endocytic-dependent mechanism, and it would be interesting to determine whether this is also through a Deltex-dependent route [41]. Both human and mouse DTX1 act, like activated Notch, to suppress the activity of the basic helix-loop-helix protein E47, a transcription factor that regulates early B-cell development. The mechanism was not defined but proposed to be independent of the CSL transcription factor [42,43].

DTX1 has also been reported to function in the nucleus to either mediate or suppress NOTCH1 activity through interaction with the NOTCH1 ICD and transcriptional coactivators. For example, overexpression of DTX1 was found to inhibit differentiation of the MNS-70 neuroprogenitor-like cell line [44]. In these cells, DTX1 inhibited transcriptional activation of MASH1 (mammalian achaete scute homologue-1), thus reproducing the phenotype observed in cells overexpressing NOTCH1. A fraction of DTX1 was found to be localised to the nucleus where it interacted and inhibited the activity of the transcriptional coactivator protein p300 [44]. This activity was proposed to mediate a CSL-independent form of Notch activity and was found to require the RING Finger ubiquitin ligase domain of DTX1.

In contrast, DTX1 has also been proposed to block canonical NOTCH1 signalling during thymocyte differentiation. Notch signalling has a key role in directing common lymphoid progenitors towards the T cell fate, at the expense of B cell development [45,46,47]. Overexpression of DTX1 in haematopoietic stem cells inhibited thymocyte development, while the B cell population was significantly increased compared to controls [48]. It was proposed that the interaction between DTX1 and NOTCH1 ICD in the nucleus blocked recruitment of transcriptional coactivators required to activate expression of NOTCH1 downstream targets through CSL. Unlike the case with the MNS-70 neuroprogenitor-like cell line [44], the N-terminal WWE repeats of DTX1 alone were able to suppress Notch ICD activity.

DTX1 also has Notch-independent activities. For example, DTX1 targets the hypoxia-inducible factor-1α (HIF-1α) for degradation, which helps regulate T-cell activation and maintain the stability of regulatory T cells, thereby influencing immune tolerance [49]. This activity required the RING finger domain but did not involve direct ubiquitination of HIF-1α by DTX1. Instead, binding of DTX1 promoted proline hydroxylation of HIF-1α and subsequent proteosome mediated degradation following recruitment of Hippel–Lindau (VHL)-containing E3 ubiquitin ligase complex [49]. DTX1 also targets mitogen-activated protein kinase kinase 1 (MEKK1 or MAP3K), which can negatively regulate T-cell activation [50]. Moreover, DTX1 targets the apoptosis regulator cFLIP (FADD-like IL-1β-converting enzyme-inhibitory protein) for degradation through its trafficking to the lysosome to promote apoptosis in gastric cancer cells. In this case DTX1 appears to act as a tumour suppressor and downregulation of DTX1 is therefore associated with poorer prognosis [51].

### 3.3. DTX2

There has been little research on DTX2 interactions with Notch proteins. A recent study put forward evidence of NOTCH1 and DTX2 interacting to inhibit Wnt signalling by inhibition of cytoplasmic β-catenin [52] through an endocytic-dependent process. DTX2 expression has been identified in embryonic and adult heart tissue [53]. Although its function has not been linked to Notch in this tissue, dysregulated Notch signalling is important for many aspects of cardiovascular disease [54], and has been shown to regulate postnatal myogenesis [55]. DTX2 has, however, been found to inhibit myogenic differentiation independently of Notch signalling [56]. Knockout DTX2 mutant mice were found to have increased myogenic differentiation and enhanced skeletal muscle repair after injury. DTX2 suppresses the expression of MYOD (myoblast determination protein 1) by binding and monoubiquitinating JMJD1C (Jumonji Domain Containing 1C), which inhibits its demethylase activity. The subsequent enrichments of Histone3 K9 methylation at the MYOD gene locus inhibits the latter’s expression [56]. DTX2 was also found to bind and ubiquitinate several DNA repair enzymes including PARP1 (Poly(ADP-Ribose) Polymerase 1), XRCC5, XRCC6 (X-Ray Repair Cross Complementing 5 and 6) and SPT16. These interactions required binding of DTX2 to ADP-ribosylated forms of the substrate through the DTC domain. The consequences of DTX2 on DNA repair activity were not determined, however [35].

### 3.4. DTX3

It was previously thought that DTX3 does not affect Notch signalling due to the lack of WWE domains [43]. Recently, however, it was shown that DTX3 is able to ubiquitinate and promote NOTCH2 degradation in oesophageal cancer cells, leading to decreased proliferation [57]. Interestingly, only DTX3 and DTX3L (both lacking WWE domains) out of all of the human DTX proteins were able to bind to NOTCH2, indicating specialisation of interactions between different Notch and DTX proteins.

Independently of Notch, DTX3 was found to suppress epithelial-mesenchymal transition (EMT) in papillary thyroid carcinoma, and this was linked to ubiquitination and down-regulation of XRCC5, which, in addition to its DNA repair functions, promotes the AKT (AK strain transforming) signalling pathway and epithelial-mesenchymal transition (EMT) [58]. DTX3 may act as a tumour suppressor in other cancers since XRCC5 has also been found to be expressed in gastric cancer, breast cancer and hepatocellular carcinoma [58].

### 3.5. DTX3L

Like DTX3, DTX3L lacks the WWE domains that are involved in binding to the Notch ankyrin regions. However, it has been shown that DTX3L can act as a heterodimer with DTX1 to downregulate NOTCH1 and promote sprouting of lymphatic vessels [59]. Heterodimerisation of DTX1 and DTX3L occurs through their respective N-termini and this augments DTX3L-mediated auto-ubiquitination [32]. Acting as a heterodimer, DTX3L downregulated Notch signalling and DTX3L knockout mice displayed increased levels of activated NOTCH1 ICD. The mechanisms by which DTX3L/DTX1 down regulated NOTCH1 were not investigated, however, and so it is not known if this mechanism involves endocytic regulation of full-length NOTCH1 or whether the target was ICD after its proteolytic release. The dimerization of DTX proteins provides an extra layer of regulation that may fine-tune the distinct functions of the DTX paralogues within different cellular contexts.

Independently of Notch, DTX3L has been found to form a heterodimer with PARP9 and act to promote Interferon (IFN) signalling as part of an antiviral response by binding to STAT1 (Signal Transducer And Activator Of Transcription 1) and ubiquitinating Histone H2BJ [60]. The complex also targets viral 3C proteases for degradation providing antiviral protection both through manipulation of host responses and by directly targeting the pathogen [60]. Other studies have linked DTX3L to Histone 4 ubiquitination and DNA damage responses [61]. Intriguing recent work has investigated cross talk between ADP-ribosylation and ubiquitination post-translational modifications through DTX3L. The PARP9-DTX3L heterodimer was found to ADP-ribosylate Ubiquitin, which suppressed its catalytic transfer to target substrates, thus down regulating the E3 activity of DTX3L [62].

### 3.6. DTX4

DTX4 has been reported to act positively on NOTCH1 signalling [63]. It was found that NOTCH1 was ubiquitinated at the plasma membrane, which triggered its dynamin-mediated endocytosis. It was proposed that DTX4 activates ligand-dependent signalling by promoting separation of the S1 cleaved heterodimer allowing the bulk of the ECD to transendocytose into the ligand-bearing cell. Following this event, the remaining membrane bound portion of the receptor was observed to be internalised into the signal-receiving cell and colocalised with the disintegrin-like metalloproteinase ADAM10, one of the main effectors of the NOTCH1 site 2 cleavage. Colocalization of the receptor with ADAM10 generated a membrane-tethered ICD intermediary product required for subsequent ICD release by gamma-secretase [63]. It is notable that the proposed DTX4-promoted mechanism differs from previously proposed models of ligand-dependent Notch activation in which endocytic-generated pulling force, instead of dissociating the S1 cleaved heterodimer, partially unfolds the NRR region to expose the S2 site to ADAM10 dependent cleavage [64,65]. In this model, the latter occurs whilst Notch is still at the cell surface. The two models are not mutually exclusive, however, and it is possible that DTX4 regulates which activation mechanism occurs according to cell context.

DTX4, like DTX1, also serves a Notch-independent role in the maintenance of immune activity by regulating type 1 IFN signalling. DTX4 is recruited by NLR Family Pyrin Domain Containing 4 (NLRP4) to mediate the proteasomal degradation of the Serine/threonine-protein kinase (TBK1), thereby restricting the induction of type 1 IFN following viral infection [66]. Typically, viral infection activates TBK1, which leads to type 1 IFN signalling after IRF3 (IFN regulatory factor 3) phosphorylation, but abnormal production of type 1 IFN can lead to autoimmune disorders, as a result of aberrant immune responses [66]. Therefore, the recruitment of the NLRP4-DTX4 complex is involved in both innate and adaptive immunity.

DTX4 also promotes adipogenic differentiation during an early phase in the pathway called mitotic clonal expansion [67]. A stable knockdown of DTX4 via recombinant shRNA lentivirus in vitro led to decreased expression of adipocyte promoting transcription factors Peroxisome proliferators activated receptor γ (PPAR-γ) and CCAAT/enhancer binding protein α (C/EBP α). DTX4 shRNA knockdown also led to increased expression of Wnt signalling genes, which are also inhibitors of adipocyte differentiation [67]. Since increased adipocyte levels relate to obesity then targeting DTX4 might provide a therapeutic strategy to alleviate this condition.

### 3.7. Regulation of DTX Proteins in Development and Disease

There have been few reports of developmental phenotypes using animal models. Some functional redundancy between genes may account for limited studies in vivo. Lehar and Bevan (2006) created DTX1 and DTX2 double mutant mice and induced additional DTX4 knockdown in cell culture, through RNAi. DTX1/DTX2 knockout mice did not present any clear abnormalities in the development and maturation of either B or T cells. Bone marrow from DTX1 knockout mice was able to compete with bone marrow from wild-type mice when transplanted into irradiated recipient mice and give rise to normal populations of lymphocytes [68]. Although the three DTX homologs could inhibit Notch signalling in vitro, no such difference was observed in double DTX1/DTX2 knockout mice. However, the addition of the DTX4 RNAi knockdown potentiated Notch-mediated lineage commitment in double mutants, and thymocyte expansion was slightly impaired [68]. These data corroborate an earlier report that progression through different stages of the thymocyte maturation process involves an alternative Notch pathway with DTX1 downregulating later but not early stages of the lineage [69].

DTX proteins have been found to have an active role in development by promoting the differentiation of progenitor cells to certain cell fates. One way to regulate DTX-induced cell differentiation is through modulating its expression. For example, DTX1 expression is positively regulated by NF-kB (Nuclear Factor kappa-light-chain-enhancer of activated B cells) in marginal zone B-lymphocyte development and also in mouse mesencephalic neural crest cells, promoting gliogenesis [70,71]. Work in zebrafish found HER2 and HER8a (Hairy/Enhancer of split homologues) to directly bind to N-boxes within the DTX1 promoter suppressing its transcription [37]. This downregulation of DTX1 likewise resulted in a reduction in neural and glial differentiation. In mammalian osteosarcoma cells, the direct binding of HES1 (Hairy/enhancer of split 1) to these N-boxes within the DTX1 promoter also inhibits DTX1 transcription and leads to an increase in cell invasion [72]. Whereas, in mouse bone marrow progenitor cells, expression of the transcription factor GATA3 was found to drive DTX1 transcription to inhibit NOTCH1 activation and arrest T cell differentiation at an early stage [73]. It is also thought that GATA3 acts together with other cofactors in order to regulate DTX1 expression [73]. Other studies have shown that DTX1 expression is activated by NOTCH1 activity suggesting a feedback loop may be involved in Notch regulation [74,75]. Regulation of DTX1 and DTX3L expression has been found to mediate shear stress dependent activation of lymphatic sprouting [59]. In this example the expression of the DTX genes was found to respond to mechanotransduction-regulated dimerisation of the transcription factor KLF2 (Kruppel Like Factor 2) with PROX1 (Prospero Homeobox 1). DTX1 and DTX3L then act as a heterodimer to down regulate NOTCH1 activity and enhance sprouting. Mutant mice lacking DTX3L have increased Notch activity and display sprouting defects [59].

Transcriptional regulation of DTX proteins has also been linked with various diseases. Expression of DTX3L transcription along with its regulatory protein BAL1 (B-aggressive lymphoma 1) is induced by IFN-γ signalling in diffuse large B-Cell lymphomas and high expression is associated with poor prognosis [76]. DTX3L expression downstream of IFN signalling has also been implicated in antiviral response mechanisms through ADP-ribosylation of viral proteins when in a heterodimer with PARP9, also an IFN stimulated gene. Reversal of DTX3L/PARP9 mediated ADP-ribosylation by the SARS-CoV-2 Nsp3 (Nonstructural protein 3) macrodomain plays a role in suppression of host viral defence. This activity of Nsp3 is a potential drug target for SARS-CoV-2 therapy [77]. DTX1 has also been implicated in viral response mechanisms by modulating the T-cell response to human immunodeficiency virus (HIV). T-cells exposed to HIV-pulsed dendritic cells have an increased expression of DTX1 [78]. This overexpression was linked to the activation of the p38/MAPK pathway, which then activates NFAT (Nuclear Factor of Activated T-cells) [78,79]. NFAT then directly binds to the promoter of DTX1 inducing its expression and thus, blocking T-cell activation and inducing T-cell anergy, an immunotolerance mechanism. Mice lacking the DTX1 gene displayed increased T-cell activation, systemic inflammation and autoimmunity [80].

DTX expression can also be regulated post transcriptionally. DCAF13 (DDB1 and CUL4 associated factor 13) is known to bind to an AU-rich element within the 3′ untranslated region of DTX3 mRNA, promoting mRNA decay [81]. This leads to an increase in NOTCH4 activation, while overexpression of DTX3 led to reduced NOTCH4 protein levels. This is likely to be physiologically relevant as DCAF13 overexpression is linked to poor prognosis in triple negative breast cancer [81]. In another example it was shown that DTX4 mRNA is negatively regulated by the microRNA, LET-7a [82]. In obstructive nephropathy, LET-7a is dysregulated leading to increased DTX4 expression, which was linked to fibrosis in ureteropelvic junction obstruction [82]. In cerebral arteriovenous malformation (AVM) a reduction of the expression of N6-methyladenosine methyltransferase (METTL3) was found to result in an increase in Notch activity in endothelial cells and defective angiogenesis [83]. In normal conditions, METT3L modifies and stabilised DTX3L mRNA with N6-methyladenosine, and DTX3L then acts to down regulate NOTCH1 by forming a heterodimer with DTX1.

Post translational modification of DTX proteins may also play a role in their regulation. All DTX paralogues contain numerous post-translational modification sites encompassing phosphorylation, acetylation, methylation, ubiquitination and sumoylation [36]. However, most modification sites were discovered through high throughput methods and the effect on DTX regulation and/or function have thus, not been experimentally tested. One identified post translational regulator of DTX is TANK-binding kinase 1 (TBK1) which phosphorylates DTX3L on serine 9 [84]. This kinase is thought to act downstream of Ras signalling and is known to stimulate innate immune responses by phosphorylating the transcription factors NF-kB and IRF3 [84,85]. TBK1 is aberrantly activated in various human cancers, however, and it would be interesting to determine if the TBK1-mediated phosphorylation of DTX3L is involved in driving cancer cell survival [84]. Interestingly, DTX4 targets TBK1 for degradation through K48-linked polyubiquitination on K670 after being recruited by an adaptor protein NLRP4 (NLR Family Pyrin Domain Containing 4) to regulate the IFN-mediated pathogen response pathway [66]. Interestingly, DTX1 can be targeted for degradation by a Su(dx) homologue, AIP4 (Atrophin-1-interacting protein 4) also known as ITCH [86]. AIP4 polyubiquitinates DTX1 with K29-linked ubiquitin, which has been connected to the lysosomal degradation of DTX1 [86]. It is hypothesised that the WW domain in AIP4 may directly interact with a PPLP motif found in the proline-rich region of DTX1 [86,87]. In *Drosophila*, Dx can additionally be targeted for degradation by Nedd4 in a Notch-dependent manner [18].

## 4. Vertebrate Su(dx)-Related Proteins

### 4.1. Domain Structure of Su(dx)-Related Proteins

**Figure 3:**
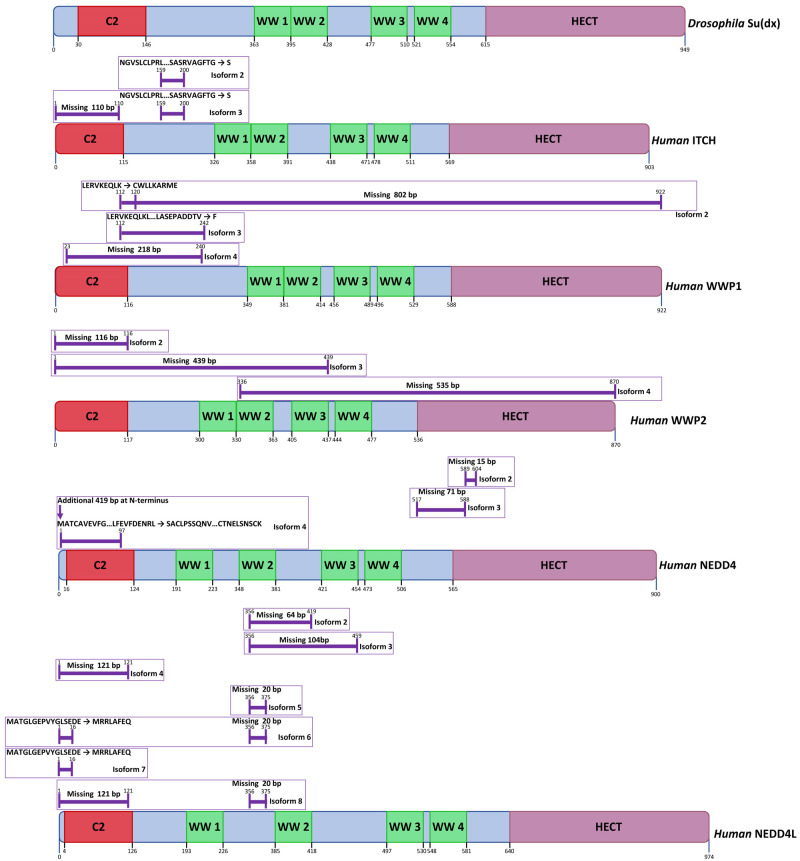
Human Su(dx)-related protein isoforms. Su(dx)-related proteins belong to the NEDD4 family of E3 ubiquitin ligases which share a conserved catalytic HECT domain at the C-terminus, WW domains which bind to Proline rich sequences, in particular PPXY motifs, and an N-terminal C2 domain which mediates interactions with membrane lipids. Human NEDD4 family members that have been documented to interact with Notch proteins are shown. Isoform variations arising from alternative splicing are indicated with purple lines and affect domain number and spacing (https://www.uniprot.org, accessed on 18 October 2021) [33].

Su(dx)-related proteins belong to the NEDD4 family of E3 ubiquitin ligases which are defined by an N-terminal C2 domain, up to 4 WW domains, and a C-terminal HECT domain (Figure 3). The C2 domain is a phospholipid-binding motif, which mediates interaction between NEDD4 family proteins and the cell membrane [88]. WW domains, named after conserved tryptophan residues, are motifs that bind to proline-rich peptides, most notably PPXY sequences, which may be located in direct targets for ubiquitination, or in adaptor proteins that bridge NEDD4 family proteins with their target [89]. HECT domains are E3 ubiquitin ligase domains that catalyse the transfer of ubiquitin to a target substrate. This is achieved via a covalent intermediate comprising a thioester linkage between ubiquitin and a conserved catalytic cysteine near the C-terminus of the HECT domain. Ubiquitin is first passed to the catalytic cysteine domain from an E2 ubiquitin ligase, which binds to the HECT domain at an N-terminal subdomain known as the N-lobe. Subsequently, the ubiquitin is passed to the target substrate through covalent linkage to a lysine side chain, or to a previously linked ubiquitin to form a polyubiquitinated product [90]. The domain structures of members of the human NEDD4 family proteins that have been linked to Notch regulation are shown in Figure 3. Alternative splicing generates several isoforms that vary in domain composition, or domain spacing, which may affect target specificity or the HECT domain activity (Figure 3). Most notably, all of the human NEDD4 family members that are depicted in Figure 3 have isoforms that lack the N-terminal C2 domain, which may alter the subcellular localisation of the protein and in some cases promote ubiquitin ligase activity, since autoinhibitory interactions between the C2 domain and the HECT domain have previously been noted [91]. Certain isoforms of WWP1 and WWP2 express only N-terminal regions and lack the catalytic HECT domain. Despite this, they may exert regulatory functions on interacting proteins [92].

### 4.2. ITCH/AIP4

In mice, loss of function mutations of ITCH (also called AIP4) result in an inflammatory phenotype with immune dysfunction and systemic autoimmune disease [93]. ITCH regulates numerous aspects of immune system responses and tolerance, for example ITCH suppresses T-cell receptor activation in T-cells to raise the threshold of an immune response [94,95]. The consequences of ITCH loss of function are not confined to immune response dysregulation, as ITCH mutants also display epidermal hyperplasia and increased keratinocyte proliferation. A number of protein targets that may be involved in the above-described phenotypes are ubiquitinated and regulated by ITCH, including the kinase JunB, the G protein coupled receptor CXCR4, the transcription factor p63, and Notch proteins [94,96,97,98].

As with *Drosophila* Su(dx), ITCH has been found to promote Notch endocytosis and degradation independently of the presence of Notch ligands [99]. Mouse ITCH was found to directly bind to NOTCH1 through its WW domains and ubiquitinate the NOTCH1 ICD when coexpressed in Jurkat cells. This interaction occurred despite NOTCH1 lacking any PPXY motif, which is the normal motif through which WW domain proteins bind to their substrate. Deletion mapping showed that the N-terminal region of ICD, including the RAM/ankyrin region, was involved in this non-canonical WW domain interaction [98]. However, other works have indicated that adapter proteins play a role in bringing NOTCH1 and ITCH into a complex. The Notch endocytic regulator NUMB has been found to act as one such adaptor and binds both NOTCH1 and ITCH to promote Notch ubiquitination and degradation [100]. Like Su(dx), the NUMB/ITCH complex was found to be involved in endosomal sorting of NOTCH1 through to the late endosome for lysosome-mediated degradation [101]. NUMB regulation of NOTCH1 through ITCH was found to depend on the cell polarity protein SHOOTIN1. The latter promoted the ubiquitination and degradation of NUMB by a RING domain ubiquitin ligase LNX1/2 (Ligand of NUMB Protein X1 and X2), and also directly bound to ITCH and inhibited the latter’s ability to ubiquitinate NOTCH1 [102]. Interestingly, in cell line-based assays, while NUMB expression promoted ITCH-mediated degradation of NOTCH1 and promoted muscle cell differentiation, it did not affect NOTCH3, indicating specificity of interactions between different Notch orthologues [103]. A complex of α-arrestin (ARRDC1) and β-arrestin has also been found to act as an adaptor and promote ITCH-dependent ubiquitination of NOTCH1, with ARRDC1 binding to ITCH WW domains through PPXY motifs [104]. NOTCH2 degradation is also promoted by ITCH [105]. In addition, AIP4/ ITCH polyubiquitinates DTX1 with K29-linked ubiquitin and this promotes the lysosomal degradation of DTX1 [86]. A similar interaction has been reported in *Drosophila* [18].

### 4.3. NEDD4

As in *Drosophila* [15,18], mammalian NEDD4 (also known as NEDD4-1) downregulates Notch activity. Full-length NOTCH1 was found to be targeted for poly-ubiquitination by NEDD4 in muscle C2C12 cells, and in vivo, loss and gain of function of NEDD4 was associated, respectively, with increased or decreased NOTCH1 protein levels in rat muscle tissue [106]. Interestingly, NEDD4 expression levels were found to increase and NOTCH1 to decrease during muscle atrophy that resulted specifically from lack of use, but not when atrophy resulted from other causes such as starvation, or in diabetic models [106]. In another study NEDD4 expression was found to be promoted by the transcription factor IFN regulated factor 4 (IRF4). Loss of IRF4, and subsequent loss of NEDD4 expression, was found to be associated with up-regulated NOTCH2 expression and signalling during progression of chronic lymphocytic leukaemia [107]. NEDD4 has also been linked to cell fate regulation through degradation of NOTCH1 during embryonic development of hematopoietic stem cells [108]. In this case, activation of the G-protein coupled receptor (GPR183), and its subsequent internalisation into cells, recruits β-arrestin1. The complex acts to promote NOTCH1 degradation through NEDD4-dependent ubiquitination. This regulatory link between GPR183 and NOTCH1 was found to be conserved between zebrafish and mammalian cells.

### 4.4. NEDD4-like (NEDD4-L)

Although previously thought to target mostly ion channel proteins rather than signalling pathways [109], NEDD4L (also known as NEDD4-2) has been linked to NOTCH1 down regulation in breast cancer cell lines, and high NEDD4L expression is associated with longer breast cancer relapse-free periods [109]. Interestingly, NEDD4L downregulation in breast cancer was found to be caused through the action of a microRNA (miR-106b-25) [110]. In heart development, NEDD4L was also found to be regulated by another microRNA (mir-10) although in this case the relevant NEDD4L target proteins were not identified. In *Drosophila*, regulation of Notch by miR-1 expression occurs by the targeting of NEDD4 expression [111].

### 4.5. WWP1

WWP1 is a NEDD4 family protein whose altered expression has been associated with both oncogenic and tumour-suppressive functions in a number of cancers, including osteosarcoma, gastric cancer, prostate cancer, melanoma, oral cancer and breast cancer [112,113]. These associations with various cancers are likely mediated by a number of different identified WWP1 target proteins but the link with Notch signalling has been little studied. Previous work using pull down assays has shown, however, that NOTCH1 ICD can participate in a complex with WWP1 [114]. Furthermore, WWP1 can colocalise with NOTCH1 in early endosome locations when expressed in C2C12 cells. WWP1 can be localised in both cytoplasmic and nuclear locations, but colocalisation with NOTCH1 results in depletion of WWP1 from the nucleus, potentially affecting regulation of downstream nuclear targets of WWP1 activity [114].

### 4.6. WWP2

WWP2, like WWP1, has been associated with a number of different cancers, acting as either an oncogene or as a tumour suppressor in different cell contexts. WWP2 has been particularly linked to the regulation of NOTCH3 [115]. This interaction was first detected through a proteomics study for proteins binding to the NOTCH3 ICD. WWP2 was found to bind directly to NOTCH3 through a PPXY motif (a motif exclusive to NOTCH3 amongst the human Notch proteins), ubiquitinating NOTCH3 and promoting its degradation through lysosomal trafficking, similar to *Drosophila* Su(dx) [115]. Interestingly, in ovarian high grade serous carcinoma, around 77% of the tumours had deletions of the WWP2 gene, indicating a link between WWP2 down regulation and ovarian cancer. Over-expression of WWP2 in ovarian cancer cell lines resulted in cell cycle arrest [115]. WWP2 has also been found to promote NOTCH3 degradation in hepatic cells [116].

DISHEVELLED, a Wnt pathway component, has been found to bind to and promote WWP2 activity, indicating a possible mechanism of cross talk between the Wnt and NOTCH3 pathways [117]. DISHEVELLED binds to the WW domains of WWP2 through an interaction with a PPXY motif. The N-terminal WW1 and WW2 domains of WWP2 were the preferred biding sites. In addition, an interaction between DISHEVELLED and the C2 domain of WWP2 promoted WWP2 activity, possibly by removing an autoinhibitory interaction between the C2 domain and the HECT domain. Similar relief of intra-molecular inhibitory interactions has been observed for other NEDD4 family protein. Blocking of autoinhibitory interactions by tyrosine phosphorylation of NEDD4 family proteins has also been observed [118,119,120].

### 4.7. Therapeutic Targeting of Su(dx)-Related Proteins

Numerous links between Su(dx)-related proteins and disease have indicated that this family of proteins might be considered therapeutic targets, for example to control tumour progression and metastasis. A number of inhibitors have been shown to directly or indirectly suppress activity of NEDD4 family proteins. Curcumin is a polyphenol agent, derived from turmeric, which inhibits NEDD4 activity indirectly by suppressing its expression and enhancing its degradation [121]. This inhibits pancreatic tumour cell proliferation and invasion, and enhances apoptosis. The latter is likely due to increased PTEN activity, as the latter is a target for NEDD4 polyubiquitination. However, consequences on Notch were not investigated in this study [121]. Diosgenin, a steroidal saponin, was found to also have similar effects on NEDD4 in prostate cancer cells [122].

Other studies have identified inhibitors that directly bind to the HECT domain of WWP2 to block its ubiquitin ligase activity [123]. A small molecule inhibitor heclin, was found to bind to the HECT domains of NEDD4-family proteins and promote oxidation of the catalytic cysteine to form cross-linked disulphide bonded dimers, inhibiting ubiquitin ligase activity [124]. Other computational modelling and structural studies have identified and optimised agents that act through cysteine covalent modification [125,126].

Indole-3-carbinol (I3C) is another molecule that inhibits ubiquitin ligase activity of NEDD4 proteins by direct binding to the HECT domain. Treatment of melanoma cells with I3C was found to suppress their proliferation [126,127]. I3C inhibition of WWP1 was also found to reactivate the tumour suppressor PTEN and suppress MYC-dependent growth promoting activities in prostate cancer cells that had high WWP1 expression [128]. Interestingly, inhibition of the HECT E3 ligase of WWP1 and NEDD4 by I3C prevents the ubiquitination of the SARS-CoV-2 spike protein. As with a number of other viruses, the latter modification regulates lysosomal trafficking of the virus and promotes viral budding, making NEDD4/WWP1 potential therapeutic target for COVID19 treatment [129].

Small molecule agonists of NEDD4 family proteins may also have therapeutic applications. In hepatic cells, WWP2 targets NOTCH3 and this down regulatory activity suppresses fibrosis. WWP2 activity is limited by the binding of a protein phosphatase, PPM1G. Disruption of this inhibitory complex by a naturally occurring compound costunolide [116] leads to upregulation of WWP2 activity on NOTCH3. Downregulation of NOTCH3 after costunolide treatment has been found to account for its anti-hepatic fibrosis properties [116]. Another compound, N-acetylcysteine, has been found to promote NOTCH2 lysosomal-dependent degradation through increasing ITCH ubiquitin ligase activity when applied to glioblastoma multiforme tumour cells. The activity of N-acetylcysteine, which was independent of its anti-oxidant properties, was found to inhibit cancer cell proliferation and tumour growth in vitro and in vivo, making it a promising approach towards treatment of a cancer that has few effective alternative clinical approaches [105].

## 5. Conclusions

A growing interest in the regulation of Notch proteins through endocytosis and trafficking is highlighting the roles of vertebrate Dx and Su(dx)-related proteins in both positively and negatively regulating Notch (summarised in Table 1). While not always essential for viable development, the importance of these regulatory proteins in the regulation of cell fate in adult organisms, particularly in the immune system and myogenesis is becoming more evident. Numerous disease conditions have been linked to misregulation of Dx and Su(dx)-related proteins, and loss and gain of function of different members have been linked to several cancers. The relatively limited viability requirements of these proteins in normal cells makes them attractive targets for suppressing tumours in situations where cancer cells have become dependent on their activity. The increasing understanding of the regulation of Dx and Su(dx)-related proteins in vertebrate models holds considerable promise for the development of novel therapeutic approaches.

## Figures and Tables

**Table 1:** Summary of regulatory interactions between Dx and Su(dx)-related proteins and Notch.

Species	Homologuegue	Effect onEffect on Notch Signalling	Function	References
*Drosophila melanogaster*	Dx	Upregulates	Binds Notch ICD, promotes clathrin-dependent endocytosis of the receptor from the plasma membrane Stabilizes the receptor on endosome membrane to facilitate ligand-independent activation by ADAM 10-independent mechanism.	[16,17,20,25]
		Downregulates	Promotes Notch removal from plasma membrane and downregulates ligand-dependent Notch activation.	[17]
Vertebrates	DTX1	Upregulates	Enhances transcription of Notch target genes through interaction with NICD	[34]
		Downregulates	Inhibits recycling of the receptor back to the plasma membrane to down regulate ligand-dependent signalling.	[38]
			Inhibits Notch coactivator recruitment.	[48]
	DTX2	Not determined	Required for endocytosis-dependent downregulation of β-catenin by NOTCH1	[52]
	DTX3	Downregulates	Enhances NOTCH2 receptor degradation	[57]
	DTX3L	Downregulates	Heterodimerizes with DTX1 to downregulate NOTCH1.	[59]
	DTX4	Upregulates	Triggers endocytosis of the NOTCH1 receptor and activates ECD shedding.	[63]
*Drosophila melanogaster*	Su(dx)	Upregulates	Promotes Notch clathrin-independent endocytosis. When HECT domain is inactive, Su(dx) promotes ligand-independent signalling by ADAM10-dependent mechanism.	[17]
		Downregulates	Promotes Notch clathrin-independent endocytosis, and, when HECT domain is active, Su(dx) promotes receptor-ubiquitination and degradation, to downregulate ligand-dependent and ligand-independent signalling.	[13,15,17]
	Nedd4	Downregulates	Promotes Notch endocytosis, Ubiquitinates and destabilizes Notch.	[15,18]
Vertebrates	ITCH/AIP4	Downregulates	Promotes endocytosis and directs the receptor towards lysosome-mediated degradation.	[101,105]
	NEDD4	Downregulates	NEDD4-mediated ubiquitination is necessary and sufficient for Notch1 down-regulation.	[106]
	NEDD4-L	Downregulates	NEDD4-L promotes NOTCH1 ubiquitination and degradation.	[109,110]
	WWP1	Not determined	Colocalises with Notch1, which inhibits WWP1 localisation to nucleus.	[113,114]
	WWP2	Downregulates	Promotes Notch3 degradation.	[115,116]

## Data Availability

Not applicable.
